# Crystal structure of 3-ethynyl­benzoic acid

**DOI:** 10.1107/S2056989015016515

**Published:** 2015-09-12

**Authors:** Chiara Venturini, Nicolas Ratel-Ramond, Andre Gourdon

**Affiliations:** a CEMES–CNRS BP 94347, 29 Rue J. Marvig, 31055 Toulouse, France

**Keywords:** crystal structure, 3-ethynyl­benzoic acid, hydrogen bonding

## Abstract

In the title compound, C_9_H_6_O_2_, the carb­oxy­lic acid group is almost in the plane of the benzene ring, making a dihedral angle of 2.49 (18)°. In the crystal, mol­ecules are linked by pairs of O—H⋯O hydrogen bonds, forming classical acid–acid inversion dimers, with an *R*_2_^2^(8) ring motif. The dimers are linked by pairs of C—H⋯O hydrogen bonds forming chains, enclosing *R*_2_^2^(16) ring motifs, propagating along the *c*-axis direction.

## Related literature   

For the potential applications of terminal alkynes in crystal engineering, see: Dai *et al.* (2004 ▸). For the synthesis of the title compound, see: Bischoff *et al.* (2008 ▸). For the NMR spectrum of the title compound, see: Bleisch *et al.* (2014 ▸). For other syntheses of the title compound, see: Jones *et al.* (2008 ▸); Pawle *et al.* (2011 ▸). For the crystal structure of the 4-ethynyl benzoic acid methyl ester, see: Dai *et al.* (2004 ▸).
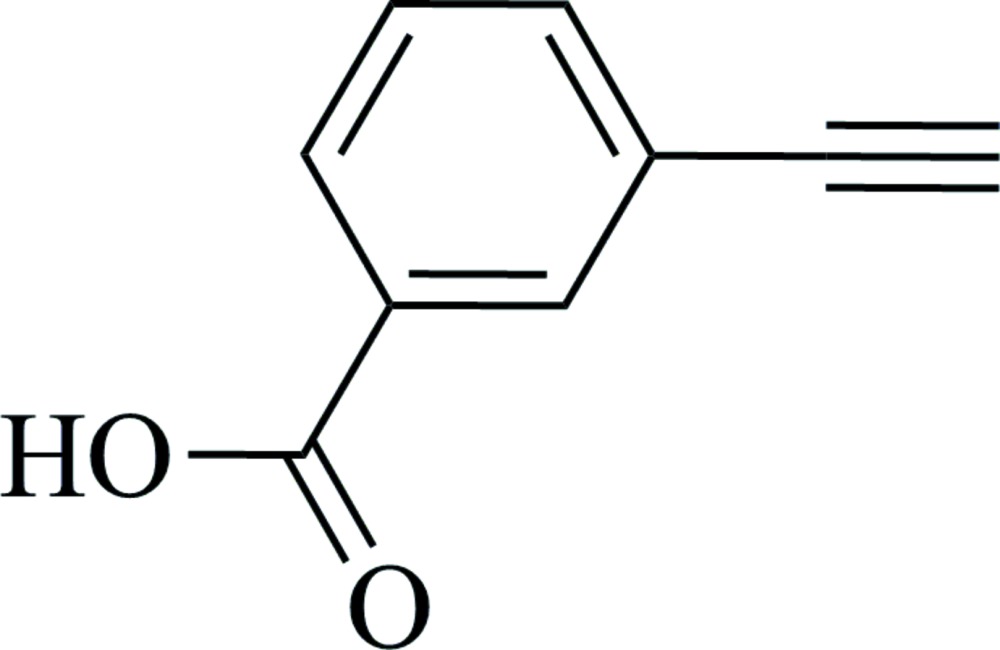


## Experimental   

### Crystal data  

C_9_H_6_O_2_
*M* *_r_* = 146.14
Triclinic, 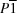

*a* = 3.8630 (7) Å
*b* = 8.3000 (9) Å
*c* = 11.7490 (1) Å
α = 101.44°
β = 93.8°
γ = 99.83°
*V* = 361.84 (8) Å^3^
*Z* = 2
Mo *K*α radiation
μ = 0.10 mm^−1^
*T* = 293 K
0.9 × 0.4 × 0.1 mm

### Data collection  

Bruker Kappa APEXII CCD diffractometer
Absorption correction: multi-scan (*SADABS*; Bruker, 2012 ▸) *T*_min_ = 0.664, *T*_max_ = 0.745
2719 measured reflections
1317 independent reflections
1086 reflections with *I* > 2σ(*I*)
*R*_int_ = 0.012

### Refinement  

*R*[*F* ^2^ > 2σ(*F* ^2^)] = 0.037
*wR*(*F* ^2^) = 0.117
*S* = 1.08
1317 reflections
124 parameters
All H-atom parameters refined
Δρ_max_ = 0.13 e Å^−3^
Δρ_min_ = −0.13 e Å^−3^

### 

Data collection: *APEX2* (Bruker, 2012 ▸); cell refinement: *SAINT* (Bruker, 2012 ▸); data reduction: *SAINT*; program(s) used to solve structure: *SIR2011* (Burla *et al.*, 2012 ▸); program(s) used to refine structure: *SHELXL97* (Sheldrick, 2008 ▸); molecular graphics: *Mercury* (Macrae *et al.*, 2008 ▸); software used to prepare material for publication: *WinGX* (Farrugia, 2012 ▸) and *PLATON* (Spek, 2009 ▸).

## Supplementary Material

Crystal structure: contains datablock(s) global, I. DOI: 10.1107/S2056989015016515/su5200sup1.cif

Structure factors: contains datablock(s) I. DOI: 10.1107/S2056989015016515/su5200Isup2.hkl

Supporting information file. DOI: 10.1107/S2056989015016515/su5200Isup3.cml

**Fig. 1.**
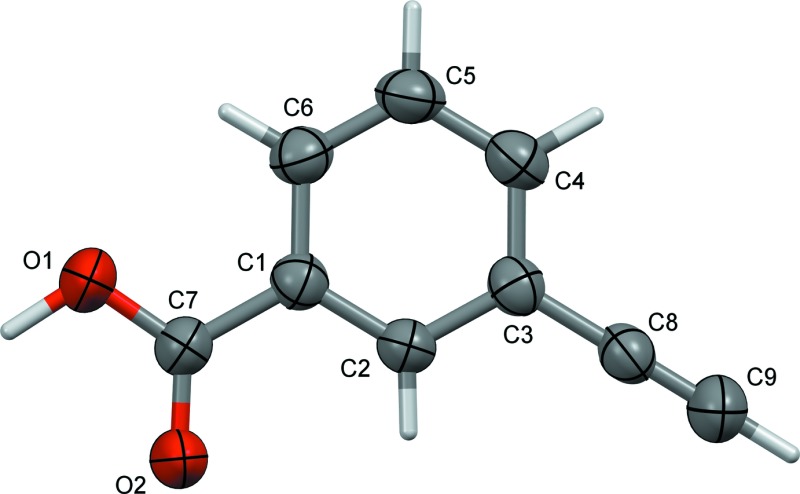
The molecular structure of the title compound, with atom labelling. Displacement ellipsoids are drawn at the 50% probability level.

**Fig. 2.**
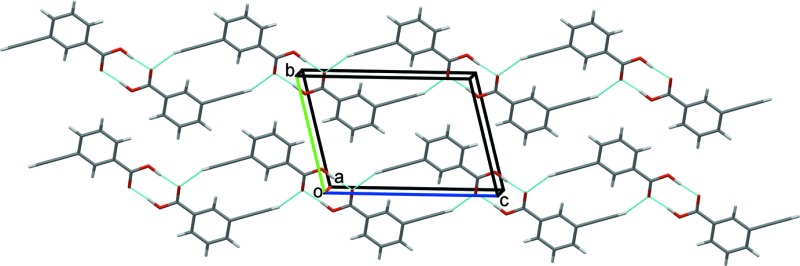
A view along the a axis of the crystal packing of the title compound. The hydrogen bonds are shown as dashed lines (see Table 1).

. DOI: 10.1107/S2056989015016515/su5200fig1.tif
The mol­ecular structure of the title compound, with atom labelling. Displacement ellipsoids are drawn at the 50% probability level.

a . DOI: 10.1107/S2056989015016515/su5200fig2.tif
A view along the *a* axis of the crystal packing of the title compound. The hydrogen bonds are shown as dashed lines (see Table 1).

CCDC reference: 1422308

Additional supporting information:  crystallographic information; 3D view; checkCIF report

## Figures and Tables

**Table 1:** Hydrogen-bond geometry (, )

*D*H*A*	*D*H	H*A*	*D* *A*	*D*H*A*
O1H1O2^i^	1.03(2)	1.59(2)	2.625(2)	175(2)
C9H9O2^ii^	0.96(2)	2.50(2)	3.386(2)	153(2)

Symmetry codes: (i) 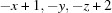; (ii) 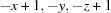.

## supplementary crystallographic information

### S1. Chemical context

In recent years, the inter­est in compounds with an alkyne C≡CH bond has increased due to their versatility in coupling reactions such as Glaser-Hay or Sonogashira. At the same time the crystallography of terminal alkynes has become an intense field of study for the potential applications in crystal engineering (Dai *et al.*, 2004). Additionally, the presence of a carboxyl­ate group on these compounds makes them potential candidates for the formation of metal organic frameworks *viz.* MOFs. With these applications in mind, we have synthesized the title compound and report herein on its crystal structure.The synthesis of 4-ethynyl­benzoic acid has been reported previously (Jones *et al.*, 2008; Pawle *et al.*, 2011), but not its crystal structure. Only the crystal structure of the ester, 4-ethynyl­methyl­benzoate, has been described previously (Dai *et al.*, 2004).

### S2. Synthesis and crystallization

3-ethynyl­benzoic acid is commercially available. In this work it was obtained by saponification and acidification of the corresponding ester in methanol/water with lithium hydroxide, following the reported procedure (Bischoff *et al.*, 2008). Vapor diffusion of dichlo­methane/hexane afforded light yellow crystals. The NMR spectrum is in agreement with the literature values (Bleisch *et al.*, 2014): ^1^H-NMR (300 MHz, DMSO) 1H 13.22 (1H, s, O—H), 7.94-7.97 (2H, m, H6; H2), 7.73-7.71 (1H, m, H3), 7.53 (1H, t, J = 7.5 Hz, H7), 4.30 (1H, s, CHh). ^13^C-NMR (75 MHz, DMSO) 13C 166.4 (Ci), 135.8 (Cd), 132.2 (Ca), 131.3 (Ch), 129.7 (Cb), 129.2 (Cc), 122.1 (Ce), 82.5 (Cf), 81.69 (Cg).

### S2.1. Refinement

Crystal data, data collection and structure refinement details are summarized in Table 2. All of the H atoms were located in difference Fourier maps and freely refined.

### Crystal data

**Table:**

C_9_H_6_O_2_	*Z* = 2
*M**_r_* = 146.14	*F*(000) = 152
Triclinic, *P*1	*D*_x_ = 1.341 Mg m^−^^3^
Hall symbol: -P 1	Mo *K*α radiation, λ = 0.71073 Å
*a* = 3.8630 (7) Å	Cell parameters from 1127 reflections
*b* = 8.3000 (9) Å	θ = 2.6–26.1°
*c* = 11.7490 (1) Å	µ = 0.10 mm^−^^1^
α = 101.44°	*T* = 293 K
β = 93.8°	Parallelepiped, colourless
γ = 99.83°	0.9 × 0.4 × 0.1 mm
*V* = 361.84 (8) Å^3^	

### Data collection

**Table:**

Bruker Kappa APEXII CCD diffractometer	1317 independent reflections
Radiation source: sealed tube	1086 reflections with *I* > 2σ(*I*)
Graphite monochromator	*R*_int_ = 0.012
phi and ω scans	θ_max_ = 26.2°, θ_min_ = 3.4°
Absorption correction: multi-scan (*SADABS*; Bruker, 2012)	*h* = −3→4
*T*_min_ = 0.664, *T*_max_ = 0.745	*k* = −10→10
2719 measured reflections	*l* = −14→14

### Refinement

**Table:**

Refinement on *F*^2^	0 constraints
Least-squares matrix: full	Hydrogen site location: difference Fourier map
*R*[*F*^2^ > 2σ(*F*^2^)] = 0.037	All H-atom parameters refined
*wR*(*F*^2^) = 0.117	*w* = 1/[σ^2^(*F*_o_^2^) + (0.0649*P*)^2^ + 0.035*P*] where *P* = (*F*_o_^2^ + 2*F*_c_^2^)/3
*S* = 1.08	(Δ/σ)_max_ < 0.001
1317 reflections	Δρ_max_ = 0.13 e Å^−^^3^
124 parameters	Δρ_min_ = −0.13 e Å^−^^3^
0 restraints	

### Special details

**Table:**

Geometry. Bond distances, angles etc. have been calculated using the rounded fractional coordinates. All su's are estimated from the variances of the (full) variance-covariance matrix. The cell esds are taken into account in the estimation of distances, angles and torsion angles

### Fractional atomic coordinates and isotropic or equivalent isotropic displacement parameters (Å^2^)

**Table:**

	*x*	*y*	*z*	*U*_iso_*/*U*_eq_	
O1	0.3420 (4)	0.18714 (15)	1.00233 (9)	0.0714 (5)	
O2	0.4634 (3)	−0.00243 (13)	0.85678 (9)	0.0648 (4)	
C1	0.2847 (3)	0.24041 (16)	0.81364 (11)	0.0432 (4)	
C2	0.3024 (3)	0.18897 (16)	0.69499 (12)	0.0428 (4)	
C3	0.2316 (3)	0.29147 (16)	0.61941 (12)	0.0434 (4)	
C4	0.1418 (4)	0.44533 (18)	0.66466 (14)	0.0509 (5)	
C5	0.1251 (4)	0.49579 (18)	0.78270 (14)	0.0558 (5)	
C6	0.1965 (4)	0.39454 (18)	0.85767 (13)	0.0509 (5)	
C7	0.3696 (4)	0.13271 (17)	0.89319 (12)	0.0477 (4)	
C8	0.2570 (4)	0.23974 (16)	0.49657 (12)	0.0489 (5)	
C9	0.2828 (5)	0.1985 (2)	0.39633 (14)	0.0629 (6)	
H1	0.421 (7)	0.110 (3)	1.054 (2)	0.133 (9)*	
H2	0.362 (4)	0.086 (2)	0.6622 (13)	0.050 (4)*	
H4	0.088 (4)	0.519 (2)	0.6115 (14)	0.063 (4)*	
H5	0.058 (4)	0.601 (2)	0.8124 (14)	0.069 (5)*	
H6	0.188 (4)	0.429 (2)	0.9398 (16)	0.065 (5)*	
H9	0.302 (5)	0.164 (2)	0.3144 (19)	0.087 (6)*	

### Atomic displacement parameters (Å^2^)

**Table:**

	*U*^11^	*U*^22^	*U*^33^	*U*^12^	*U*^13^	*U*^23^
O1	0.1157 (10)	0.0684 (7)	0.0382 (6)	0.0396 (7)	0.0105 (6)	0.0108 (5)
O2	0.1041 (9)	0.0569 (7)	0.0434 (6)	0.0361 (6)	0.0138 (5)	0.0146 (5)
C1	0.0424 (7)	0.0445 (7)	0.0430 (7)	0.0091 (5)	0.0044 (5)	0.0095 (5)
C2	0.0462 (8)	0.0391 (7)	0.0442 (7)	0.0111 (5)	0.0051 (5)	0.0090 (5)
C3	0.0393 (7)	0.0462 (7)	0.0459 (7)	0.0082 (5)	0.0036 (5)	0.0129 (5)
C4	0.0517 (8)	0.0463 (8)	0.0580 (9)	0.0138 (6)	0.0011 (6)	0.0165 (6)
C5	0.0599 (9)	0.0448 (8)	0.0634 (10)	0.0201 (6)	0.0027 (7)	0.0060 (7)
C6	0.0524 (8)	0.0512 (8)	0.0484 (8)	0.0155 (6)	0.0052 (6)	0.0041 (6)
C7	0.0566 (8)	0.0479 (7)	0.0399 (7)	0.0139 (6)	0.0064 (6)	0.0084 (6)
C8	0.0526 (8)	0.0485 (8)	0.0510 (9)	0.0148 (6)	0.0052 (6)	0.0189 (6)
C9	0.0827 (12)	0.0647 (10)	0.0483 (9)	0.0250 (8)	0.0116 (8)	0.0176 (7)

### Geometric parameters (Å, º)

**Table:**

O1—C7	1.2902 (18)	C4—C5	1.376 (2)
O2—C7	1.2415 (18)	C5—C6	1.378 (2)
O1—H1	1.03 (2)	C8—C9	1.175 (2)
C1—C2	1.3845 (19)	C2—H2	0.938 (16)
C1—C6	1.389 (2)	C4—H4	0.992 (16)
C1—C7	1.4735 (19)	C5—H5	0.959 (17)
C2—C3	1.3907 (19)	C6—H6	0.955 (18)
C3—C4	1.393 (2)	C9—H9	0.96 (2)
C3—C8	1.437 (2)		
			
C7—O1—H1	112.4 (13)	O2—C7—C1	121.61 (12)
C2—C1—C7	119.56 (12)	O1—C7—C1	116.24 (13)
C6—C1—C7	120.24 (12)	C3—C8—C9	179.04 (17)
C2—C1—C6	120.17 (12)	C1—C2—H2	122.5 (9)
C1—C2—C3	120.08 (12)	C3—C2—H2	117.4 (9)
C2—C3—C8	120.12 (12)	C3—C4—H4	119.9 (9)
C4—C3—C8	120.72 (13)	C5—C4—H4	119.6 (9)
C2—C3—C4	119.15 (13)	C4—C5—H5	119.5 (10)
C3—C4—C5	120.48 (14)	C6—C5—H5	120.1 (10)
C4—C5—C6	120.39 (14)	C1—C6—H6	119.3 (10)
C1—C6—C5	119.73 (14)	C5—C6—H6	121.0 (10)
O1—C7—O2	122.16 (13)	C8—C9—H9	179.5 (19)
			
C6—C1—C2—C3	−0.05 (19)	C6—C1—C7—O2	−176.98 (14)
C7—C1—C2—C3	−178.37 (12)	C1—C2—C3—C4	−0.26 (19)
C2—C1—C6—C5	0.3 (2)	C1—C2—C3—C8	178.71 (12)
C7—C1—C6—C5	178.59 (14)	C2—C3—C4—C5	0.3 (2)
C2—C1—C7—O1	−178.90 (13)	C8—C3—C4—C5	−178.63 (14)
C2—C1—C7—O2	1.3 (2)	C3—C4—C5—C6	−0.1 (2)
C6—C1—C7—O1	2.8 (2)	C4—C5—C6—C1	−0.2 (2)

### Hydrogen-bond geometry (Å, º)

**Table:**

*D*—H···*A*	*D*—H	H···*A*	*D*···*A*	*D*—H···*A*
O1—H1···O2^i^	1.03 (2)	1.59 (2)	2.625 (2)	175 (2)
C9—H9···O2^ii^	0.96 (2)	2.50 (2)	3.386 (2)	153 (2)

Symmetry codes: (i) −*x*+1, −*y*, −*z*+2; (ii) −*x*+1, −*y*, −*z*+1.
