# Supplementary figures and images for: Crystal structure of 3-ethynyl­benzoic acid
Source: Acta Crystallogr E Crystallogr Commun. 2015 Sep 12;71(Pt 10):o750–1. doi: 10.1107/S2056989015016515 (PMC4647427; doi:10.1107/S2056989015016515)

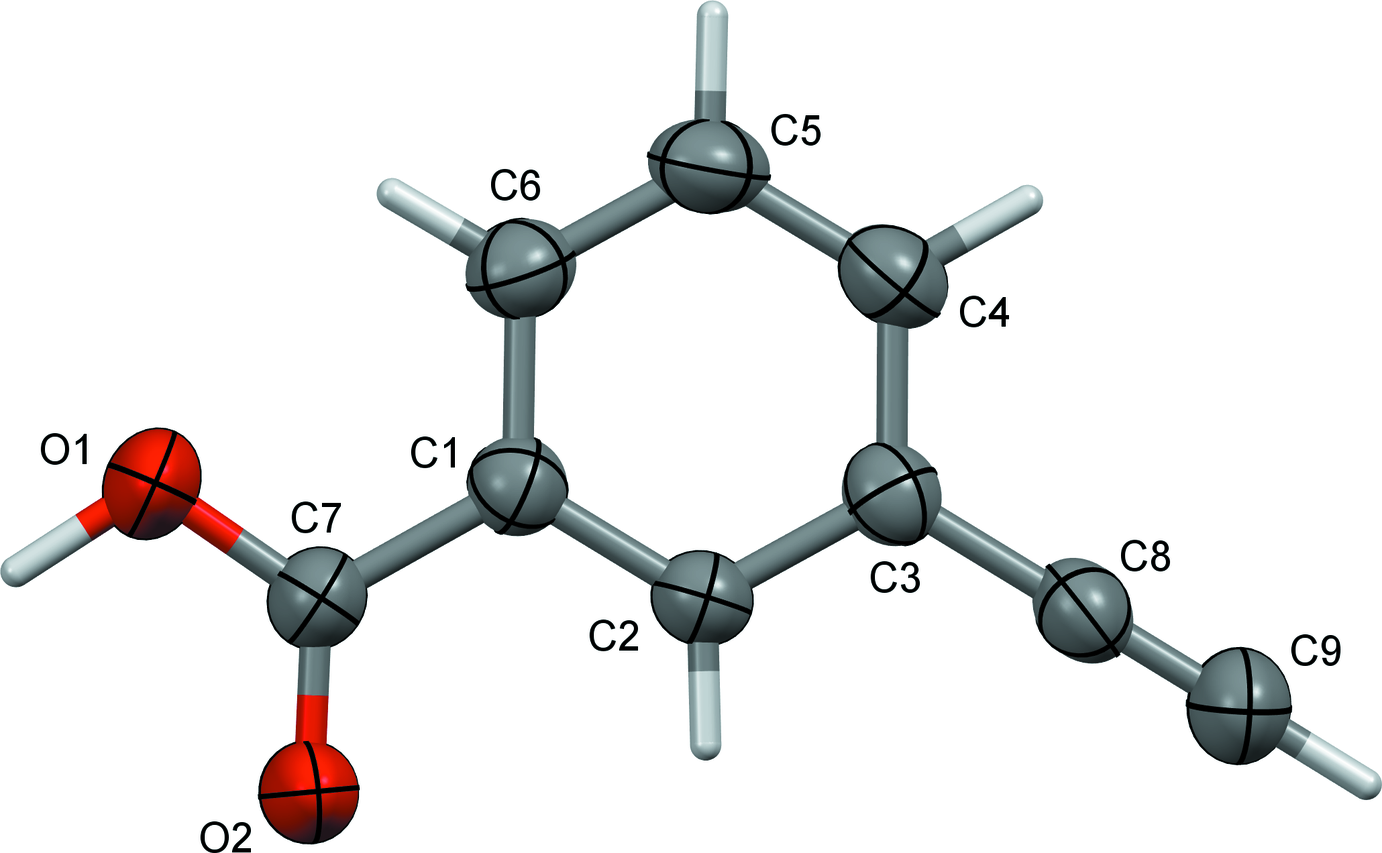

Supplement: Supplementary file 4 [file e-71-0o750-fig1.tif]

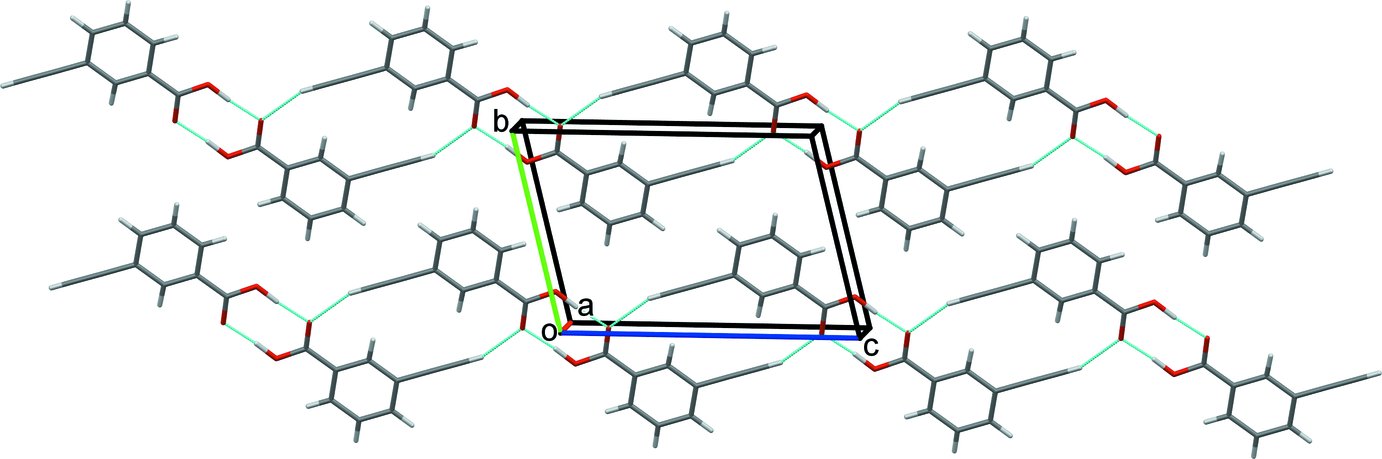

Supplement: Supplementary file 5 [file e-71-0o750-fig2.tif]
